# Racial and ethnic disparities in chronic health conditions among women with a history of gestational diabetes mellitus

**DOI:** 10.34172/hpp.2021.08

**Published:** 2021-02-07

**Authors:** Shahrzad Bazargan-Hejazi, Maria Ruiz, Shakir Ullah, Gazala Siddiqui, Maria Bangash, Shahbaz Khan, Wendy Shang, Parissa Moradi, Magda Shaheen

**Affiliations:** ^1^Department Psychiatry, College of Medicine, Charles R. Drew University of Medicine and Science and UCLA David Geffen School of Medicine, Los Angeles, CA, USA; ^2^College of Medicine, Charles R. Drew University of Medicine and Science, CA, USA; ^3^UCLA David Geffen School of Medicine, Los Angeles, CA, USA; ^4^Khyber Medical College, Pakistan, & College of Medicine at Charles Drew University of Medicine and Science, CA. USA; ^5^Department of Obstetrics and Gynecology, University of Texas at Houston, Texas, USA; ^6^Southern California University of Health and Sciences, CA, USA; ^7^Ayub Medical College, Pakistan; ^8^College of Science and Health, Biomedical Science, Charles R. Drew University of Medicine and Science, Ca, USA; ^9^Department of Obstetrics and Gynecology, Charles R. Drew University of Medicine and Science, Ca, USA

**Keywords:** Female, Diabetes, Gestational, Body mass index, African Americans, Chronic condition, Smoking, Disparity

## Abstract

**Background:** This study aims to examine and determine the role of race/ethnicity in chronic conditions in women diagnosed with gestational diabetes mellitus (GDM) during any of their previous pregnancies.

**Methods:** We used the National Health and Nutrition Examination Survey (NHANES) from2007–2016 to identify women who self-reported prior GDM and chronic disease diagnoses such as cardiovascular disease, hypertension, depression, and type 2 diabetes mellitus (T2DM).We used bivariate analysis using the chi-square test (χ²) and multiple logistic regressions to perform statistical test for associations, taking into consideration design and sample weight.

**Results:** Among participants with prior GDM diagnoses, black women had a 74.4% prevalence of chronic disease, followed by Whites, 58.5% Hispanics, 58.0%, and Asians, 51.9% (P=0.009).Black women with prior GDM diagnoses had 2.4 odds of having chronic conditions compared to Whites (adjusted odds ratio [AOR]=2.40, 95% confidence interval [CI] = 1.28-4.50). In addition, they had higher odds of being former smokers (AOR=1.73, 95% CI=1.01-2.96),current smokers (AOR=1.96, 95% CI=1.06-3.61), having a body mass index (BMI) of 25-29.9(AOR=2.55, 95% CI=1.10-5.87), or a BMI ≥30 (AOR=4.09, 95% CI = 2.05-8.17) compared to their White counterparts. Hispanic women had lower odds of being diagnosed with GDM and associated chronic diseases.

**Conclusion:** Black women with GDM were disproportionally affected and at higher risk to be diagnosed with chronic conditions. Smoking and obesity were strongly associated with chronic disease diagnoses. Our findings also suggest a ‘Hispanic Paradox’, requiring further study. These findings inform primary care clinicians and Obstetricians, and Gynecologists of at-risk patients who could benefit from lifestyle modification recommendations and counseling.

## Introduction

Gestational diabetes mellitus (GDM) is one of the most prevalent pregnancy complications.^1^ About 14% of pregnancies worldwide are affected by GDM representing approximately 18 million births annually.^2^ In the United States, GDM occurs in about 6% of pregnancies.^3^ GDM is defined as hyperglycemia that is first detected during pregnancy.^2^ Based on the American College of Obstetricians & Gynecologists (ACOG) recommendations, pregnant women should be screened for GDM at 24 to 28 weeks of gestation with an oral glucose tolerance test (OGTT).^4^ The OGTT determines an individual’s ability to handle glucose load after a meal. It can demonstrate a person’s ability to metabolize a standardized measured amount of glucose and involves taking multiple blood samples over time.^5^ The test is done by first measuring a pregnant woman’s fasting blood glucose, followed by administrating a 50 g glucose solution. If, after 1 hour, the blood glucose level is abnormal or >140 mg/dL, it is recommended to perform a 3-hour OGTT by administering a 100g oral glucose solution. Women with two or more abnormal values are diagnosed with gestational diabetes.^6^ Target glucose values in women with GDM are ≤ 95 mg/dL with fasting, ≤ 140 mg/dL 1 hour postprandial, or ≤ 120 mg/dL 2 hours postprandial.^7^ A body mass index (BMI) of 25 kg/m^2^ or greater plus an additional risk factor (e.g., physical inactivity, a first-degree relative with diabetes, high-risk ethnicity, previous GDM, hypertension, a previous baby with birth weight >4000 g, polycystic ovarian syndrome (PCOS ) warrants early screening, preferably at the initiation of prenatal care.^8^

Risk factors for GDM, which consistently emerge in the literature, include overweight/obesity,^9^ excessive gestational weight gain,^10^ ethnicity,^11^ advanced maternal age,^12^ family and personal history of GDM,^13^ and other diseases of insulin resistance, such as PCOS.^14^ GDM is also a risk factor for maternal and neonatal complications, including increased risk of macrosomia, shoulder dystocia, respiratory distress syndrome, neonatal hypoglycemia, hyperbilirubinemia, birth defects, stillbirth and subsequent childhood and adolescent obesity.^15^ Additional pregnancy complications include preterm birth,^16^ development of pre-eclampsia,^17^ increased risk of cesarean delivery, and developing type 2 diabetes mellitus (T2DM).^18^ About 60% of women with GDM will develop diabetes within 10 years of delivery.^19-21^ It is also evident that women with a history of GDM are at a higher risk of acquiring cardiovascular diseases, independent of the development of T2DM,^22,23^ hypertension, dyslipidemia, and depression compared to those without the history of GDM.^24^ In 2007, GDM increased national medical costs by $636 million, i.e., $596 million for maternal costs and $40 million for neonatal costs.^25^

Minority women in the United States have increased rates of GDM as well as increased rates of chronic disease.^26^ As reported, black and Hispanic patients have 1.81 (95% confidence interval [CI]: 1.13-2.89, *P*< 0.05), and 2.45 (95% CI: 1.48-4.04, *P* < 0.001) adjusted relative risk of GDM, respectively.^27^ Additionally, the age-adjusted prevalence of GDM by race-ethnicity is lowest for non-Hispanic whites (4.1%) and highest among Asian Indians (11.1%).^28^ Some ethnic groups not born in the United States may be at increased risk of GDM compared to those born in the United States; hence they may have needs for particular preventive and culturally sensitive care.^18^ However, our knowledge is limited regarding ethnic disparities among women with a prior diagnosis of GDM and subsequent chronic diseases development. Therefore, this study’s purpose is twofold: 1) to report racial/ethnic disparity in chronic conditions in women diagnosed GDM during any one of their previous pregnancies. 2) To determine the adjusted role of race/ethnicity in developing chronic disease conditions among these women. We hypothesize that minority women diagnosed with GDM will have statistically significantly higher odds of reporting chronic conditions, controlling other variables, including socio-demographics, smoking, and obesity. Our findings may benefit primary care physicians, and obstetricians and gynecologists to protect the long-term health outcomes of women diagnosed with GDM.

## Materials and Methods

### Study design and data

This was a cross-sectional study where we analyzed the National Health and Nutrition Examination Survey (NHANES) data from 2007 to 2016. The primary goal of the NHANES survey is to assess the health and nutritional status of the US residents. The program is funded by the Center for Disease Control and Prevention (CDC), and it includes a nationally representative sample of approximately 7000 US adults and children. The data is derived from participants’ survey as well as their physical and laboratory examinations. The minority population is oversampled to enhance the reliability of the statistical tests. The interviews are conducted in English and Spanish and take approximately 40 minutes to administer.^29^

### Study eligibility

For the current study, we included women 20 years of age and older who answered ‘yes’ to the following question: “During any pregnancy, were you ever told by a doctor or other health professional that you had diabetes, sugar diabetes, or gestational diabetes? Please do not include diabetes that you may have known about before the pregnancy.” We excluded female participants 19 years of age or younger as the NHANES questionnaire does not include their information in the publicly available dataset due to disclosure risks. We defined chronic disease as women who answered ‘yes’ to any of the following questions:

1. “Has a doctor or other health professional ever told you that you had a cardiovascular disease?”
2. “Has a doctor or other health professional ever told you that you had hypertension?”
3. “Has a doctor or other health professional ever told you that you had T2DM?”

Diagnosis of major depressive disorder was determined if the respondent scored five or more on the Patient Health Questionnaire (PHQ-9). We used self-reported race/ethnicity and categorized it into four groups: non-Hispanic whites, non-Hispanic blacks, Hispanics, and Asians/others. Chronic diseases examined were cardiovascular disease, hypertension, depression, and T2DM.

### Analysis plan

We used the chi-square test to assess the association between study variables and chronic conditions. We used multiple logistic regressions to test the independent association between race/ethnicity with the chronic conditions, controlling for socio-demographic variables, general health condition, smoking, and BMI. Logistic regression also allows to control for numerous confounders when using a relatively large sample size, therefore, eliminates confounding effects. All analyses were conducted, taking into consideration the design and sample weight of the NHANES study. Since less than 5% of the data had missing information, we simply eliminated the missing cases from the study analysis.^30^ We used SAS software V.9.3 (SAS Institute, Cary, North Carolina, USA) to analyze the data and considered *P* < 0.05 as statistically significant.

## Results

Our study sample consisted of 917 females who had been diagnosed with GDM in the past and had ever been diagnosed with chronic disease. The majority of the women (50.0%) were aged 20-44, and 43.9% were in the 45-64 age group. Whites comprised 62.9% of the sample, black 10.9%, Hispanics 17.4%, and Asian/others made up 8.9% of the total (Table 1). As reflected in Table 1, among participants with a prior history of GDM, black women had the highest prevalence of chronic disease (74.4%), followed by Whites (58.5%) and Hispanics (58.0%) (*P*=0.009). Moreover, developing a chronic condition was associated with socio-demographic variables and women’s general health, smoking status, and BMI (*P*=0.05). However, after adjusting for the study variables, only race/ethnicity, smoking status, and BMI remain statistically significant. Black women who had been previously diagnosed with GDM had 2.4 times higher odds of having chronic conditions relative to Whites (adjusted odds ratio [AOR] = 2.40, 95% CI = 1.28-4.50). Additionally, being a former smoker (AOR=1.73, 95% CI=1.01-2.96), current smoker (AOR=1.96, 95% CI=1.06-3.61), having a BMI of 25-29.9 (AOR=2.55, 95% CI=1.10-5.87), and a BMI ≥30 (AOR=4.09, 95% CI = 2.05-8.17) were also associated with having a higher odds of chronic health conditions in women who were previously diagnosed with GDM *(*Table 2).

## Discussion

Our finding that black women with GDM are disproportionally affected and have increased risk of being diagnosed with any chronic conditions compared to other racial/ethnic groups is alarming, considering personal and economic burdens associated with these conditions.^25,31,32^ This finding is consistent with others’ reporting in which non-Hispanic black women had a higher risk of developing diabetes than the other racial-ethnic groups.^27,33^ Additionally, we found that former and current smokers, and women with a BMI of 25 or higher, had an increased risk of developing chronic health conditions. This finding is consistent with others showing a positive relationship between increasing BMI and chronic disease prevalence.^34^ Furthermore, the CDC guidelines report that those who smoke are more likely to develop chronic conditions such as heart diseases, stroke, and lung cancer than nonsmokers.^35^ However, due to data limitation, our primary outcome, i.e., chronic conditions, was too general, limiting our ability to offer any specific intervention. For example, the associations between GDM with T2DM,^19,36^ hypertension,^37,38^ depression,^39^ and cardiovascular disease,^22,40^ have been reported in empirical studies. Given the elevated risk of GDM in black women, it is important to identify factors that increase their risk of developing chronic conditions such as cardiovascular disease.

In our study, being a GDM diagnosed Hispanic woman was not associated with ever been diagnosed with a chronic disease, compared to previous studies.^27^ The lower prevalence of GDM or diabetes among Hispanics has been referred to ‘Hispanic Paradox’, in which immigrants who become very sick tend to return to their native country.^28^ Additionally, as reported, 50% of Hispanic patients with gestational diabetes will develop diabetes five years after delivery.^41^

Primary care clinicians and obstetricians, and gynecologists can recommend lifestyle modification to women, specifically black women, with a history of GDM. More specifically, they can counsel these patients about the benefits of adding regular exercise to their routines, maintaining a healthy diet, and refraining from smoking to prevent the development of chronic conditions later on in life. Many minority women do not have access to health care after their delivery as they lose health insurance.^42^ Identifying this subset of high-risk women and educating them at their post-partum visit can decrease the risk of chronic disease. In the current COVID-19 pandemic context, many patients have been reluctant to visit their primary care physicians or the emergency department. Crowded hospitals may be hotspots of transmission for COVID-19. Therefore, many healthcare providers have been offering telemedicine services.^43^ In the current situation, it is all the more critical to educate at-risk patients regarding lifestyle modifications to prevent chronic diseases since diagnosis and monitor of chronic condition via telecommunication devices could be more difficult and lead to underdiagnoses of these conditions.^44^

The use of large national data strengthens our study. Our study is among a few studies that, using population-based data, focus on the role of race/ethnicity in women with a history of GDM in developing chronic conditions. However, NHANES data is cross-sectional; therefore, the main limitation is the inability to determine the temporal sequence of exposure and outcome. However, the most logical explanation for the observed result is that diagnoses of chronic conditions proceed with race/ethnicity. We also consider that our secondary data analysis findings could have been influenced by the potential sources of error associated with sampling, data measurement, non-response, or missing data in NHANES data. We were aware of these limitations and maximized our effort to elicit accurate results by considering the design and sample weight of the NHANES study and eliminating the missing cases from the study analysis since they were less than 5%.^45^

## Conclusion

Black women with GDM were disproportionally affected and were nearly 2.5 times more likely to be diagnosed with a certain chronic conditions compared to other racial/ethnic groups. We further found that former and current smokers and women with a BMI 25 or greater had an increased likelihood of developing chronic health conditions. Other studies with larger samples are needed to stratify GDM across specific chronic diseases. The findings of such studies could be used to design and implement public health measures that could prevent morbidity and mortality in black women diagnosed with GDM.

## Acknowledgments

The research team would like to acknowledge the administrative support provided by Kaveh Dehghan, MBA.

## Funding

Research for this article was supported in part by NIH Accelerated Excellence in Translational Sciences (AXIS) grant number 2U54MD007598-07; and the University of California at Los Angeles (UCLA) Clinical and Translational Science Institute (CTSI), grant number UL1TR001881.

## Competing interests

The authors report no conflicts of interest in this work.

## Ethical approval

Not Applicable.

## Authors’ contributions

MR: Principle investigator; study concept and design; interpretation of data; drafting of the manuscript. BH: Study concept and design; interpretation of data; leading and finalizing the manuscript acquisition. SU, GS, MB, SK, WS, PM: Critical revision of the manuscript; interpretation of the data. MS: Data acquisition; data analysis; interpretation of the data.

**Table 1:** Characteristics of sample with gestational diabetes mellitus (N = 917)

**Characteristic**	**Total n = 917** **(weighted %)**	**With chronic diagnosis** **(weighted %)**	**No chronic diagnosis** **(weighted %)**	***P*** ** value**
Age				<0.001
20-44	470 (50.0%)	250 (50.2%)	220 (49.8%)	
45-64	380 (43.9%)	271 (66.0%)	109 (34.0%)	
65+	67 (6.1%)	61 (89.9%)	6 (10.1%)	
Race/ethnicity				<0.008
White	344 (62.9%)	216 (58.5%)	128 (41.5%)	
Black	168 (10.9%)	130 (74.4%)	38 (25.6%)	
Hispanic	293 (17.4%)	183 (58.0%)	110 (42.0%)	
Asian/other	112 (8.9%)	53 (51.9%)	59 (48.1%)	
Country of birth				<0.005
US	631 (81.0%)	430 (61.8%)	201 (38.2%)	
Other countries	286 (19.0%)	152 (50.2%)	134 (49.8%)	
Citizenship status				<0.001
US citizen	785 (91.5%)	518 (61.1%)	267 (38.9%)	
Non-US citizen	128 (8.5%)	62 (43.4%)	66 (56.6%)	
Education				<0.001
≤High school	419 (38.2%)	296 (67.8%)	123 (32.2%)	
>High school	498 (61.8%)	286 (54.4%)	212 (45.6%)	
Income				<0.001
≤200 Federal poverty level	457 (39.4%)	315 (66.7%)	142 (33.3%)	
>200 Federal poverty level	392 (60.6%)	223 (55.3%)	169 (44.7%)	
Covered by health insurance		<0.045
Yes	740 (84.5%)	478 (61.2%)	262 (38.8%)	
No	177 (15.5%)	104 (50.8%)	73 (49.2%)	
General health condition		<0.011
Fair or poor	304 (25.4%)	258 (84.5%)	46 (15.5%)	
Good/very good/excellent	613 (74.6%)	324 (51.0%)	289 (49.0%)	
Smoking status				<0.002
Never smoker	567 (58.8%)	329 (53.8%)	238 (46.2%)	
Former smoker	170 (22.0%)	120 (65.9%)	50 (34.1%)	
Current smoker	180 (19.2%)	133 (69.9%)	47 (30.1%)	
Number of pregnancies		<0.123
≤3	498 (61.4%)	293 (56.8%)	205 (43.2%)	
≥4	419 (38.6%)	289 (63.9%)	130 (36.1%)	
BMI				< 0.001
<25	175 (21.2%)	77 (36.3%)	98 (63.7%)	
25-29.9	234 (25.8%)	127 (52.7%)	107 (47.3%)	
≥30	503 (53.0%)	373 (71.9%)	130 (28.1%)	

Abbreviation: BMI, body mass index.

**Table 2:** Adjusted role of race/ethnicity in chronic disease development in a sample of women with gestational diabetes mellitus

**Characteristic**	**Odds ratio (95% CI)**	***P*** ** value**
Age		
20-44	Ref	0.001
45-64	2.18 (1.47-3.24)	
65+	21.73 (7.17-65.85)	<0.001
Race/ethnicity		
White	Ref	
Black	2.40 (1.28-4.50)	0.007
Hispanic	0.79 (0.43-1.44)	0.427
Asian/other	1.83 (0.86-3.94)	0.117
Country of birth		
US	1.34 (0.78-2.29)	0.282
Other countries	Ref	
Education		
≤High school	1.28 (0.81-2.02)	0.279
>High school	Ref	
Income		
≤200 Federal poverty level	1.13 (0.70-1.84)	0.607
>200 Federal poverty level	Ref	
Covered by health insurance		
Yes	1.67 (0.86-3.22)	0.126
No	Ref	
General health condition		
Fair or poor	5.16 (2.95-9.03)	<0.001
Good/very good/excellent	Ref	
Smoking status		
Never smoker	Ref	
Former smoker	1.73 (1.01-2.96)	0.045
Current smoker	1.96 (1.06-3.61)	0.032
BMI		
<25	Ref	
25-29.9	2.55 (1.10-5.87)	0.028
≥30	4.09 (2.05-8.17)	0.001

Abbreviation: BMI, body mass index.
